# Involvement of PAF metabolism in anaphylaxis and prevention by Rush-SOTI

**DOI:** 10.1186/2045-7022-3-S3-O23

**Published:** 2013-07-25

**Authors:** Z Guo, NM Tsuji

**Affiliations:** 1 Laboratory for Epithelial Immunobiology, RIKEN Yokohama Institute, Yokohama City, Japan; 2 Allergy and Mucosal Immune Tolerance Open Laboratory, RIKEN Yokohama Institute, Yokohama City, Japan; 3 Biomedical Research Institute, The National Institute of Advanced Industrial Science and Technology, Tsukuba City, Japan

## Background

Allergy is a critical problem of public health in developed countries. Anaphylaxis is a rapid allergic response with symptoms such as urticarial, itching, diarrhea, and even death. Since allergy is caused by the failure of tolerance induction to exogenous antigens, its establishment of tolerance is a fundamental therapy for allergic diseases. Oral tolerance is a physiological way to actively induce unresponsiveness to food antigens. However, oral immunotherapy using antigens occasionally fails to induce complete tolerance in patients that hinder the development of the therapy.

## Methods

To clarify the mechanism of the induction of allergy and oral tolerance, we have established a protocol with rush administration of oral antigen and evaluated how anaphylaxis is suppressed.

## Results

Using this rush specific oral tolerance induction (Rush-SOTI) protocol, we found that the drop of body temperature was partially blocked and recovery was accelerated. Antigen-specific IgE was not reduced by Rush-SOTI. Antagonists of both histamine and platelet-activating factor (PAF) completely prevented the drop of body temperature in anaphylactic mice. From the kinetics, it is suggested that histamine acts in the early phase while PAF functions in the late phase.We further analyze the cell population involved in the temperature drop. Depletion of Gr-1^+^ neutrophils but not mast cells partially prevent body temperature drop. Significant reduction of inflammatory cytokines was correlated to this effect.

## Conclusion

Together with the observation that he kinetics in both mice with Gr-1^+^ Cell-depletion and Rush-SOTI treatment were similar, it is suggested that Rush-SOTI prevents anaphylaxis by controlling PAF metabolism, and Gr-1^+^ neutrophils could be a potential therapeutic target.

## Disclosure of interest

None declared.

